# Function and Clinical Implications of Long Non-Coding RNAs in Melanoma

**DOI:** 10.3390/ijms18040715

**Published:** 2017-03-28

**Authors:** Georg Richtig, Barbara Ehall, Erika Richtig, Ariane Aigelsreiter, Tony Gutschner, Martin Pichler

**Affiliations:** 1 Institute of Experimental and Clinical Pharmacology, Medical University of Graz, Graz 8010, Austria; georg.richtig@medunigraz.at; 2 Department of Dermatology, Medical University of Graz, Graz 8036, Austria; erika.richtig@medunigraz.at; 3 Institute for Pathology, Medical University of Graz, Graz 8036, Austria; barbara.ehall@medunigraz.at (B.E.); ariane.aigelsreiter@medunigraz.at (A.A.); 4 Division of Hematology, Department of Internal Medicine, Medical University of Graz, Graz 8036, Austria; 5 Faculty of Medicine, Martin-Luther-University Halle-Wittenberg, Halle (Saale) 06120, Germany; 6 Division of Clinical Oncology, Department of Medicine, Medical University of Graz, Graz 8036, Austria

**Keywords:** lncRNA, skin cancer

## Abstract

Metastatic melanoma is the most deadly type of skin cancer. Despite the success of immunotherapy and targeted agents, the majority of patients experience disease recurrence upon treatment and die due to their disease. Long non-coding RNAs (lncRNAs) are a new subclass of non-protein coding RNAs involved in (epigenetic) regulation of cell growth, invasion, and other important cellular functions. Consequently, recent research activities focused on the discovery of these lncRNAs in a broad spectrum of human diseases, especially cancer. Additional efforts have been undertaken to dissect the underlying molecular mechanisms employed by lncRNAs. In this review, we will summarize the growing evidence of deregulated lncRNA expression in melanoma, which is linked to tumor growth and progression. Moreover, we will highlight specific molecular pathways and modes of action for some well-studied lncRNAs and discuss their potential clinical implications.

## 1. Introduction

Although melanoma only represents a small fraction of all skin cancer types, it is responsible for the majority of skin cancer related deaths [1]. Therefore, it is the most lethal cutaneous neoplasm. Unfortunately, melanoma incidence is rising worldwide and despite the development of new treatment options, metastatic disease in melanoma is still associated with high rates of mortality [2]. Prognoses of melanoma patients depend on the tumor thickness, ulceration and metastatic spread, with the 10-year survival varying from 93% to 39%, respectively [3]. The major risk factor for melanoma is ultraviolet (UV) radiation, which is responsible for a characteristic genetic signature [4]. Effectively mutational rate in melanoma is the highest within all cancer types and considered responsible for the success of immunotherapy [4]. The two main pathways of interest in melanoma nowadays are the PI3K/PTEN/Akt/mTOR signaling pathway and the RAS/RAF/MEK/ERK signal transduction cascade (Mitogen-activated protein kinase (MAPK) Signaling Pathway) [5]. The most common genetic mutation suitable for targeted therapy within the MAPK pathway, is located in the B-Raf proto-oncogene, serine/threonine kinase (BRAF) accounting for approximately 40–60% of all melanomas [6,7]. The mutation occurs at codon 600 of the *BRAF* gene in approximately 95% of all cases, the most common exchange being a valine to glutamic acid referred as *BRAF^V600E^* mutation [8]. The majority of *BRAF* mutations lead to a hyper-activation of the MAPK pathway, resulting in enhanced cell growth and survival [9,10]. Usually Rat sarcoma (RAS) activates Rapidly Accelerated Fibrosarcoma (RAF), which propagates down-stream signaling through MAPK/ERK Kinase (MEK) to extracellular signal-regulated kinase (ERK). When a high-kinase activity mutation in *BRAF* occurs, it can independently activate the MAPK pathway and in fact *BRAF^V600E^* has a ~500-fold increased activity compared to *BRAF^wt^* [11].

The second most common mutation found in melanoma is affecting the NRAS proto-oncogene, GTPase (*NRAS*) gene with a frequency of ~20% [7,12]. Therefore, the MAPK pathway is one of the major oncogenic drivers in melanoma. However, recent studies revealed additional mutations involved in growth and progression of malignant melanoma, e.g., KIT proto-oncogene receptor tyrosine kinase (*KIT*) gene in mucosal melanoma, telomerase reverse transcriptase (*TERT*) gene, germline cyclin dependent kinase inhibitor 2A (*CDKN2A*) gene, tumor protein p53 (*TP53*) gene, neurofibromin 1 (*NF1*) gene and others [13,14,15].

### 1.1. Therapeutic Modalities

Currently, cutaneous melanoma are classified into four subgroups: *BRAF^mt^*, *NRAS^mt^*, *NF1^mt^* and triple wild type [16,17]. Therefore, patients with metastatic diseases have three pharmacological therapeutic options: (i) immunotherapy; (ii) targeted therapy, focusing on the mutational status of melanoma cells; and (iii) conventional chemotherapy, if the first two options are not suitable or available [18,19,20,21,22].

#### 1.1.1. Targeted Therapy

##### BRAF Mutated Melanoma

There are two BRAF inhibitors, which are widely used for systemic treatment in metastatic melanoma: vemurafenib and dabrafenib [23,24]. In the BRAF Inhibitor in Melanoma 3 (BRIM-3) study, vemurafenib was more effective in *BRAF^V600E^* mutated melanoma compared to conventional chemotherapy (dacarbazine and DTIC) [21]. Similar results could be obtained when another BRAF inhibitor, dabrafenib, was used in patients with metastatic melanoma and further BRAF inhibitors (e.g., encorafenib) are now in clinical trials [25,26]. However, a major problem of targeted therapy is the emergence of receptor tyrosine kinase (RTK) mutations upon treatment with BRAF inhibitors [27].

Another common side effect of BRAF inhibitors is the development of new *BRAF^wt^* and *RAS^mt^* melanoma, which may be explained by a paradoxical activation of the MAPK pathway in *BRAF^wt^* melanoma [28,29]. To increase the efficiency of MAPK pathway blockage, MEK inhibitors have been developed in combination with BRAF inhibitors [30,31,32]. Effectively, dual inhibition has improved progression-free survival, overall survival and response rates [19,33]. Therefore, dual inhibition is now considered the standard of care in *BRAF^V600^* mutated melanoma.

##### NRAS Mutated Melanoma

Approximately twenty percent of all melanomas have a hotspot mutation in the *NRAS* gene [34]. Due to its downstream signaling to the MAPK pathway and the knowledge that BRAF inhibitors lead to enhanced growth of *BRAF^wt^* melanomas, several trials investigated the efficiency of MEK inhibition in this subset of melanoma [29]. However, the response rate to MEK inhibitors is variable, suggesting that NRAS signaling does not solely act on the MAPK pathway [35]. In the literature, new mutations in the MAPK pathway and other pathways involved in proliferation and growth have been suggested as putative escape mechanism, but it might also be possible that there are some epigenetic modifications involved [36].

##### NF1 Mutated Melanoma

The Cancer Genome Atlas proposed that *NF1* mutations should be included for classification in *BRAF^wt^*/*NRAS^wt^* melanoma, since reports were available showing that mutations in *NF1* led to hyper-activation of the MAPK pathway and were responsive to MEK inhibition [16,37,38]. On the downside, another study suggested loss of *NF1* mediates resistance to MEK inhibition (selumetinib) [39]. However, Krauthammer and colleagues showed that not all *NF1* mutated melanoma were responsive to MEK inhibition and that many *NF1* mutated melanoma had concurrent mutations in RAS related genes [40]. Therefore it seems that there might be some patients with *NF1^mt^* melanoma suitable for MEK inhibitor treatment, although further studies are necessary to elucidate the role of *NF1*.

##### KIT Mutated Melanoma

The KIT proto-oncogene receptor tyrosine kinase gene is frequently mutated in acral and mucosal melanoma as well as in melanomas of chronically sun-damaged skin [41]. *KIT* acts as a bona fide oncogene, resulting in higher cell proliferation, cell migration and progression through the activation of the MAPK pathway and the PI3K/mTOR pathway [42,43]. Imatinib-a receptor tyrosine kinase inhibitor-showed high response rates and beneficial effects in patients harboring a melanoma with mutations in the exons 11 and 13 of the *KIT* gene [44]. In addition, nilotinib, a tyrosine kinase inhibitor used in imatinib-resistant chronic myeloid leukaemia, as well as sunitinib, provided promising results in the treatment of *KIT* mutated metastatic melanoma [45,46]. However, it has to be mentioned that this is confined to a subset of an already small subpopulation of patients.

#### 1.1.2. Immunotherapy

The second pharmacological option in metastatic melanoma nowadays is immunotherapy. Boosting the immune system of patients with metastatic melanoma has a long history despite low success rates [47].

However, the first immunotherapy approved for metastatic melanoma was Ipilimumab in 2011 [48]. Ipilimumab improved the overall survival of patients with advanced melanoma (compared to dacarbazine) and showed a durable long-term survival of approximately 20% [49]. However, one of the major disadvantages of cytotoxic T-lymphocyte-associated protein 4 (CTLA-4) monoclonal antibody (mAb) was the low response rate of ~20%. A major contribution to immunotherapy was the discovery of the immune evasion mechanism, mediated through the expression of B7-H1 on the surface of melanoma cells, which was later described as programmed cell death ligand-1 (PD-L1) [50].

PD-L1 was able to suppress in vivo activated T-cells, which in turn reduced the immune response. When PD-L1 was blocked by a mAb, immune response was restored and a significant reduction in tumor size was achieved [50,51]. In 2012 nivolumab-a mAb against programmed cell death 1 (PD-1)-improved progression-free survival, overall survival, demonstrated an increased response rate and could achieve an up to 35% long term survival in patients with metastatic melanoma [52,53]. However, the response rate was raised up to 53%, when ipilimumab was added to nivolumab [54]. Although the rate of adverse side effects was rising as suspected, it was manageable [55].

Immunotherapy is now a major field of anti-cancer treatment and is evolving at high speed, with new substances constantly being tested. There are ongoing clinical trials for mAb against T-cell immunoglobulin and mucin-domain containing-3 (TIM-3), OX40 and CD73 [56,57]. However, similar to targeted therapy, a significant proportion of patients developed resistance upon treatment, whereas the mechanism(s) are still widely unknown.

## 2. Long Non-Coding RNAs

The ENCyclopedia of DNA Elements (ENCODE) project revealed that a surprisingly large fraction (70%–90%) of the human genome is transcribed into RNA. However, only 1%–3% of the transcriptome carries the blueprint for the synthesis of proteins, leaving the question whether or not the remaining non-coding RNA (ncRNA) transcripts are just “nature’s trash” [58,59].

NcRNAs can be classified into small ncRNAs (<200 nucleotides (nt)) and long ncRNAs (lncRNAs), depending on their size. Multiple types of small ncRNA (microRNAs (miRNAs), small interfering RNAs (siRNAs) and PIWI-interacting RNAs (piRNAs)) have been studied extensively, especially their effects on cancer development and their involvement in regulation of immune checkpoint pathways [60,61,62,63]. Long ncRNAs represent a highly heterogeneous group of RNAs, which have an extensive variability in their cellular effects, as well as their molecular influences. They can be classified by their length (>200 nt) and by their lack of a functional open reading frame, meaning they encompass less than 100 amino acids [64,65,66,67]. Hence, lncRNAs constitute a very heterogeneous group of RNA molecules, which allows them to cover a broad spectrum of molecular and cellular functions by implementing different modes of action [68,69,70,71]. From an evolutionary perspective, circumventing the energy intensive protein translation by regulating various processes through ncRNAs seems quite reasonable [72]. This regulation by lncRNA can occur via multiple mechanisms, which Wang et al. divided into four types, which we will discuss here briefly [73].

### 2.1. Signal LncRNA

lncRNAs are generally transcribed by polymerase II and their expression and stimulus response are very cell type specific, indicating a strong transcriptional control [74]. Since they are under transcriptional control, they can be seen as a signal able to detect the chromatin state of regulatory elements, or simply, the expression of associated genes. An interesting point to consider is that the cell saves resources and time by producing regulatory RNAs and circumventing protein translation. This category sees lncRNAs as a signal of gene expression patterns and is associated with time, location and developmental state. One lncRNA in this category, which is regulated by external stimuli—namely, DNA damage—is LincRNA-p21, which plays a key regulatory role in p53 transcriptional response. It acts as a transcriptional repressor of the p53 pathway. p53 directly regulates LincRNA-p21 expression by binding to the promotor of LincRNA-p21 [75].

### 2.2. Decoy LncRNA

One major mechanism of transcription regulation involves lncRNAs acting as decoys, by binding to various transcription factors, chromatin modifiers and other regulatory factors, to prevent them from executing their designated function [76]. One described mode of action involves lncRNAs acting as “miRNA sponges”, by sequestering and thus down-regulating respective miRNAs. Consequently target genes of these miRNAs are influenced significantly in their expression [77]. Experiments involving gene knockout of lncRNA should therefore indicate increased effects of the speculated bound effector molecule [78]. For example, depletion of Metastasis-associated lung adenocarcinoma transcript 1 (MALAT1) in tumor cells resulted in a reduction of tumorigenicity, while transient overexpression induced tumor proliferation and formation [79]. Based on studies by Tripathi et al. double knockdown of lncRNA MALAT1 and its suspected effectors with siRNA resulted in a rescue phenotype [80].

### 2.3. Guide LncRNA

Guide lncRNAs are categorized by their ability to bind proteins and afterwards direct the complex to a specific location. Gene expression changes can occur either in *cis* (on neighboring genes) or in *trans* (more distant genes), although this is impossible to predict based solely on the lncRNA sequence [81]. Mechanisms in *cis* could include a co-transcriptional chromatin change alongside the RNA polymerase, or serve as complementary targets for small regulatory RNAs. The in *trans* guidance is thought to be based upon lncRNAs binding to target DNA as heteroduplexes or RNA:DNA:DNA triplexes, or even RNA recognition sites on the surface of specific chromatin features. Whatever the mechanism, the result of lncRNA guidance is to regulate and bring about epigenetic changes in target genes [82].

This class/archetype encompasses lncRNAs such as Homebox (HOX) transcript antisense RNA (HOTAIR) and would be characterizable in knockdown lncRNA experiments by the missing functionality of the effector, due to its malfunctioning localization or even a phenotype resembling an effector knockout. In comparison to the decoy archetype, the guide archetype would exhibit an exacerbated phenotype instead of a rescue.

### 2.4. Scaffold LncRNA

Until now, it was thought that only proteins play a key role in scaffolding complexes and therefore the control of intermolecular interactions and signaling [83]. Recent evidence has shown, that lncRNAs may possess similar functions [84,85]. The lncRNA in this class is characterized by its ability to bind multiple effector molecules over specific domains, which brings them closer together and supports their specific functions, such as activation or repression of gene transcription. By furthering our understanding of these complex interactions and their regulation, we would then be able to exploit these mechanisms to influence cells according to our needs. This archetype would be characterizable in knockdown of the lncRNA by malfunctioning of the involved pathway or even a loss of function due to interference in the lncRNA-effector-scaffold assembly. Any further knockout of effectors would likely exacerbate the phenotype instead of saving it. When manipulating the specific lncRNA domains, there might be effects on different effectors and functions [73]. One scaffold lncRNA is the antisense lncRNA in INK4 locus (ANRIL), which directly interacts with polycomb repressive complexes (PRC1 and PRC2). Interaction with ANRIL interrupts transcriptional repression of the *INK4b* locus [73,86].

### 2.5. Multifunctionality and Novel Mechanisms

Classification of one lncRNA into multiple archetypes is not unlikely, as already described for cold induced long antisense intragenic RNA (COOLAIR) and Homebox A (HOXA) transcript at the distal tip (HOTTIP), which both fall into the signal as well as the guide class [87]. HOX transcript antisense RNA (HOTAIR) is also a multifunctional lncRNA transcribed in distal and posterior cells, thus being an anatomically specific signal, which is also involved in both PRC2 and Lysine-specific histone demethylase 1 (LSD1) complex assembly, therefore fitting the scaffold category and the localization of PRC2 as a guide [88].

However, ongoing research efforts will very likely identify lncRNAs with new modes of action that cannot be included in the aforementioned subtypes. For example, a new mechanism how lncRNA can affect metabolism and regulatory processes has recently been described by Liu and colleagues [89]. They demonstrated that the lncRNA neighbor of BRCA1 gene 2 (NBR2) directly targets the adenosine monophosphate-activated protein kinase (AMPK) during energy-stress periods. Furthermore, NBR2 depletion led to altered apoptosis/autophagy and unchecked cell cycling with increased tumor development in vivo [89,90].

These examples highlight the diverse and complex biological functions that can be executed or mediated by lncRNAs. Consequently, lncRNA deregulation can have a severe impact on cellular behavior and is often found in human diseases, especially cancer. In the following paragraph, we highlight examples of lncRNAs, whose expression is altered in human melanoma and which have been shown to functionally contribute to melanoma development and progression.

## 3. Long Non-Coding RNAs in Melanoma

### 3.1. ANRIL

In the well-studied *INK4* locus the interplay between ANRIL and chromatin-modifying complexes can be observed, in which ANRIL serves as a scaffold lncRNA. ANRIL was found in the *INK4B/ARF/INK4A* locus, has 19 exons and spans 126.3 kb. Due to alternative splicing, several long, short and circular isoforms of ANRIL exist [91]. ANRIL expression has been linked to several conditions, including the risk of melanoma [92,93].

**Figure 1:**
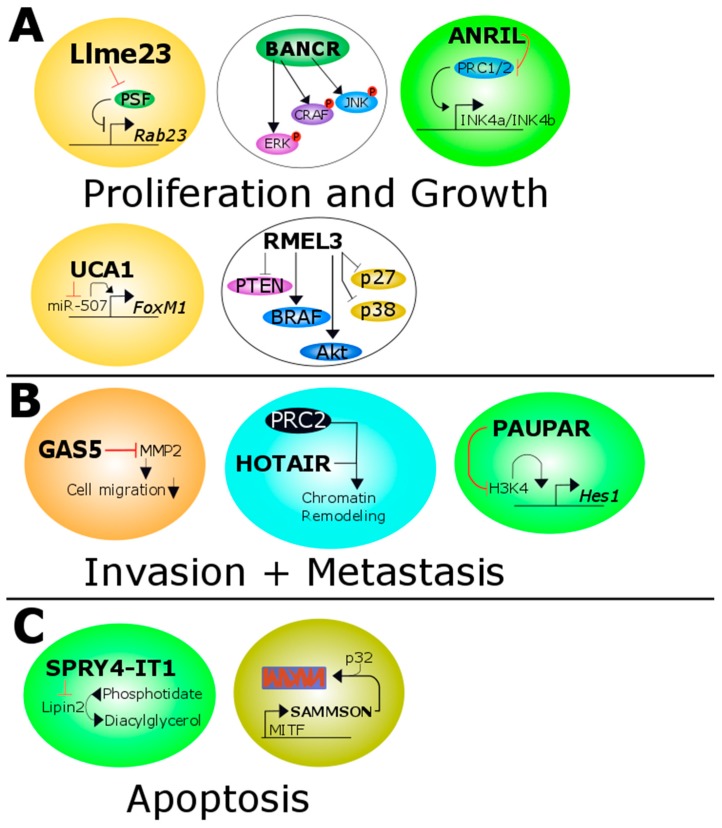
Overview of all melanoma related lncRNAs. (**A**) Llme23 binds the protein associated splicing factor which in turn can not bind to the promoter of the proto-oncogene *RAB23*. BANCR increases the activity of ERK, CRAF and JNK. ANRIL inhibits the transcription of p14/p15/p16 expression by methylation of the histone H3 of the *INK4b/ARF/INK4a* tumor suppressor locus. UCA1 targets and inactivates miR-507 and this leads to increased levels of the pro-oncogenic transcription factor FoxM1. RMel3 decreases the level of PTEN, p27 and p38 and increases the activity of BRAF and Akt; (**B**) GAS5 inhibits the matrixmetalloprotease 2 which in turn decreases the ability of migration of melanoma cells. HOTAIR leads together with PRC2 to increased chromatin remodeling. MALAT1 binds to miR-22 in cutaneous melanoma promoting MMP14 and SNAIL expression. In uveal melanoma it binds to miR-140 decreasing SLUG and ADAM10 expression. PAUPAR represses HES1 expression by inhibiting histone H3K4 demethylation; (**C**) SPRY4-IT1 inhibits Lipin2 which converts phosphatidate to diacylglycerol. SAMMSON is co-amplified with MITF and forms a complex with p32 which stabilized mitochondrial biogenesis. Red and black T shaped bars signify reduced or downregulated proteins/miRNAs or genes. Black arrows indicate overexpressed or upregulated proteins or genes. ERK: extracellular signal-regulated kinase; CRAF: C-Raf proto-oncogene, serine/threonine kinase; JNK: JUN N-terminal kinase; ANRIL: antisense lncRNA in INK4 locus; UCA1: Urothelial carcinoma-associated 1; PTEN: phosphatase and tensin homolog; BRAF: B-Raf proto-oncogene, serine/threonine kinase; GAS5: growth arrest-specific transcript 5; HOTAIR: HOX transcript antisense RNA; PRC2: polycomb repressive complexe 2; MMP14: matrix metalloproteinase 14; SNAIL: snail family transcriptional repressor; SLUG: snail family transcriptional repressor 2; ADAM10: ADAM metallopeptidase domain 10; PAUPAR: PAX6 upstream antisense RNA; SPRY4-IT1: SPRY4 intronic transcript 1; SAMMSON: Survival associated mitochondrial melanoma-specific oncogenic non-coding RNA; MITF: melanogenesis associated transcription factor.

It has been proposed that ANRIL negatively regulates *INK4b/ARF/INK4a* in *cis* through chromatin remodeling. It achieves this by binding to PRC1 and PRC2, which in turn controls lysine 27 methylation of histone H3 in the *INK4B/ARF/INK4A* tumor suppressor locus (Figure 1A) [94]. This implies that ANRIL is significantly involved in cell proliferation and furthermore in cell proliferation after DNA damage repair (see Table 1) [94,95]. Xu et al. indicated that ANRIL was overexpressed in cutaneous melanoma and uveal melanoma compared to normal tissue [96]. Knockdown of ANRIL by siRNA restored the ability of two tumor cell lines (A375 and OM431) to transcribe *INK4A* and *INK4B*. This reduced the cell’s ability to migrate and form colonies and ANRIL might therefore be a valid therapeutic target [96].

### 3.2. BANCR

BRAF-activated non-coding RNA (BANCR) is a 693nt lncRNA, which is encoded by chromosome 9 and acts as a decoy lncRNA [97]. BANCR is notable, because it is highly upregulated in human primary malignant melanoma and induced by *BRAF^V600E^* in comparison to *BRAF^wt^* melanoma. Depletion experiments demonstrated that BANCR has a regulatory function in melanoma cell migration, whereas its absence significantly decreases cellular migration. In BANCR depleted cells CXCL11 could be identified as a factor simultaneously down-regulated. When the authors supplied CXCL11 to BANCR deficient cells, CXCL11 was capable of rescuing and restoring the migratory abilities of BANCR-depleted cells [97].

Another study revealed that BANCR expression directly correlates with tumor stage and might contribute to the development of melanoma [98]. Li and colleagues demonstrated that BANCR can activate ERK1/2, its upstream molecule CRAF and JNK in-vitro and in-vivo, which led to proliferation of melanoma cells (summarized in Figure 1A and Table 1). They concluded that the link between these pathways indicates a novel regulation mechanism in melanoma proliferation [98].

### 3.3. CASC15

The lncRNA cancer susceptibility candidate 15 (CASC15) spans ~530 kb and is located on chromosome 6 between the *SOX4* and *PRL* genes [99]. It was frequently expressed in metastatic melanoma cell lines independent of their *BRAF* mutational status and was absent in normal melanocytes. Furthermore, brain metastases showed significantly higher CASC15 expression levels compared to the cutaneous cell lines [99]. Intriguingly, CASC15 expression in patient-derived FFPE samples from brain and lung metastases was found to be significantly higher compared to normal tissue and naevi. Analysis of the 10-year disease-free survival rates and CASC15 expression in stage III melanoma lymph node metastases revealed that patients with high CASC15 expression had a significantly reduced DFS. Additional in vitro experiments could show that CASC15 regulates melanoma cell phenotype switching between proliferative and invasive states (Table 1) [99].

### 3.4. GAS5

The lncRNA growth arrest-specific transcript 5 (GAS5) is located on chromosome 1 and consists of 650 bases (12 exons) [100]. Chen et al. investigated the functional role of GAS5 in melanoma and revealed that some cell lines had a reduced expression of GAS5. Moreover, GAS5-depleted cells showed a higher ability to migrate, whereas induced overexpression in such cells reduced their migratory ability and went along with decreased levels of matrix metalloproteinase (MMP) 2 protein production (Figure 1B and Table 1) [101].

### 3.5. HOTAIR

The lncRNA HOTAIR is transcribed from the *HOXC* cluster and regulates the transcription of the *HOXD* cluster (including *HOXD8*, *HOXD9*, *HOXD10*, and *HOXD11*) located on chromosome 2 [87].

HOTAIR expression was found to be significantly higher in lymph node metastases compared to primary melanoma, whereas several other lncRNAs, including MALAT1, Urothelial carcinoma-associated 1 (UCA1), and nuclear-enriched transcript 1 (NEAT1), showed no change in their expression patterns [102]. This result supports the idea that HOTAIR contributes to the metastatic behavior in melanoma, which is in line with findings from other groups that established HOTAIR as a crucial regulator of metastases in several cancer types [88,103]. In fact, HOTAIR knockdown experiments led to a decreased melanoma cell motility and invasiveness in conjunction with a reduced capability to degrade the extracellular matrix [102].

Mechanistically, HOTAIR acts as a guide lncRNA in *trans* by recruiting PRC2 to its target genes, which in turn results in H3K27 tri-methylation and an epigenetic silencing of metastasis suppressor genes (Figure 1B and Table 1) [87,104].

### 3.6. Llme23

Llme23 was found exclusively in human melanoma cell lines and it was shown to act as a decoy lncRNA, binding to the protein associated splicing factor (PSF), a known tumor suppressor [105]. Competitive binding of Llme23 to PSF prevents this negative regulatory protein from binding to the promotor of the proto-oncogene RAB23, a RAS-related small GTPase (Figure 1A and Table 1). Importantly, expression levels of RAB23 and Llme23 have been reported to be concordant [106].

### 3.7. MALAT1

MALAT1 is also known as nuclear-enriched transcript 2 (NEAT2) and has a length of ~8000 nt. It was discovered as a prognostic marker for lung cancer metastasis, although it has also been linked to multiple other human tumors [107,108]. MALAT1 knockdown resulted in impaired melanoma migration, implying possible effects on tumor dissemination. Patient-derived melanoma samples demonstrated a significantly higher MALAT1 expression in lymph node metastases, compared to the primary tumor and to adjacent tissue [109].

The molecular mode of action of MALAT1 is still not fully understood, but might involve transcriptional and epigenetic mechanisms [79,108,110]. In addition, recent studies suggest that MALAT1 may act as a competing endogenous RNA (ceRNA) by binding to tumor-suppressive miRNAs [111]. For example, MALAT1 acts as decoy lncRNA by targeting miR-22. Its depletion leads to increased cell migration and proliferation and miR-22 levels inversely indirectly correlate with MALAT1 levels. Functionally, miR-22 binds to MMP14 and SNAIL and suppresses their oncogenic function [112]. In uveal melanoma, Sun et al. could demonstrate that MALAT1 plays a similar role by targeting miR-140, which was down-regulated in tumor samples compared to normal tissue (summarized in Table 1) [113].

### 3.8. PAUPAR

The lncRNA PAX6 upstream antisense RNA (PAUPAR) was found in uveal melanoma tissues and uveal melanoma cell lines at low levels, suggesting that it might act as a tumor suppressor lncRNA [114]. Effectively it impacts tumorigenesis in vitro and in vivo by reducing tumor metastases significantly. Further experiments showed that PAUPAR acts as a guide lncRNA by inducing the silencing of the transcription factor hairy and enhancer of split-1(HES1) by inhibiting histone H3K4 tri-methylation at the *HES1* locus (Figure 1B and Table 1) [114].

### 3.9. RMEL3

RMEL3 was first described together with RMEL1 and RMEL2 by Sousa et al. who revealed that all RMEL lncRNAs were almost exclusively expressed in melanocytes and melanoma [115]. Interestingly, RMEL3 was significantly higher in *BRAF^mt^* melanoma compared to triple^wt^ (*RAS/BRAF/NF1*) melanoma and expression levels were negatively correlated with melanoma progression [115].

However, RMEL3 experiments could not confirm these clinical findings and suggested that RMEL3 plays a pro-oncogenic role, since knockdown experiments resulted in a 95% decrease of colony formation in different *BRAF^V600E^* melanoma cell lines. Additionally, MAPK and PI3K pathway activators and effectors were also impacted negatively. Multiple genes for these activators and effectors were found to be correlated with RMEL3, thus indicating the requirement of RMEL3 for MAPK and PI3K signaling (see Figure 1A). Because RMEL3 depletion decrease cell survival and proliferation in *BRAF^V600E^* melanoma cell lines, it might represent a potential therapeutic target gene in this subset of melanomas (summarized in Table 1) [116].

### 3.10. SAMMSON

Survival associated mitochondrial melanoma-specific oncogenic non-coding RNA (SAMMSON) is located 30 kb downstream of the melanoma-specific oncogene melanogenesis associated transcription factor (MITF) and is co-amplified in around 10% of all melanoma cases, although studies demonstrated that SAMMSON acts in *trans* as a decoy lncRNA by targeting p32. In melanoma, SAMMSON is the target of the melanoblast/melanoma-specific transcription factor SOX10 and its co-factor (Figure 1C). Expression of SAMMSON was detectable in over 90% of human primary melanoma and metastasis, whereas SAMMSON was undetectable in normal healthy tissue. Knockdown experiments established a role for this lncRNA in melanoma cell viability and growth, irrespective of their mutational status (*BRAF*, *NRAS*, *TP53*) [117]. By targeting SAMMSON, cell sensitivity towards MAPK-targeting therapeutics could be enhanced and MAPK-resistant cell lines were still susceptible to SAMMSON targeting (summarized in Table 1) [117].

### 3.11. SNHG5

SnoRNA host gene 5 (SNHG5) is part of the non-coding multiple small nucleolar RNA host gene family and encompasses 524 base pairs [118]. The site is known to be involved in human β-cell lymphoma [118]. Serum levels of SNGH5 were significantly increased in malignant melanoma patients of all stages compared to normal subjects and patients with squamous cell carcinoma. This indicates that SNGH5 may play a role in melanoma genesis (see Table 1) [119].

### 3.12. SPRY4-IT1

SPRY4 intronic transcript 1 (SPRY4-IT1; also known as SPRIGHTLY) is initiated in intron 1 of the *SPRY4* gene and extends to exon 3 [120]. Even though SPRY4-IT1 is located within an intron of *SPRY4*, both genes were found to be functionally and transcriptionally independent [121].

SPRY4-IT1 was shown to be over-proportionally represented in melanoma cell lines as well as in human melanoma samples compared to melanocytes [122]. It is mainly located in the cytoplasm of melanoma cells and was shown to be associated with polysomes [120,121]. Furthermore, SPRY4-IT1 reduces the abundance of the lipid phosphatase lipin 2. Hence, it may impair apoptosis, because of lipin 2-mediated alterations in lipid metabolism and the resulting lipotoxicity (see Figure 1C) [120]. Additional studies reported a decreased cell growth, invasion and differentiation, but increased apoptosis in SPRY4-IT1-depleted melanoma cells [120,121,123]. Zhao et al. demonstrated that SPRY4-IT1 expression is lower in melanocytes compared to melanoma. Interestingly, one month after ectopic overexpression of SPRY4-IT1 in melanocytes induced dendritic-like cell morphologies together with enlarged nuclei in 25% of the cells. These melanocytes were more proliferative, invasive, and formed anchorage-independent colonies, suggesting that SPRY4-IT1 plays an important role in melanomagenesis and progression [122]. Melanoma patients had significantly higher SPRY4-IT1 expression in comparison to healthy patients and furthermore higher SPRY4-IT1 levels were associated with lower overall survival as well as higher tumor stage (summarized in Table 1) [123].

### 3.13. UCA1

UCA1 was first discovered in bladder transitional cell carcinoma and so far two isoforms have been identified [124].

A study comparing the expression of six cancer-implicated lncRNAs in melanoma to their respective expression in paired adjacent healthy tissue, found elevated expression of UCA1 in melanoma, especially at advanced stages [109,125]. Knockdown of UCA1 inhibited melanoma cell migration suggest that it might be involved in tumor dissemination [109]. In fact, UCA1 might act as a decoy lncRNA targeting miR-507, an inhibitor of the pro-oncogenic transcription factor FoxM1 (Figure 1A). Depletion of UCA1 increased the levels of miR-507 and reduced FoxM1 levels which led to cell cycle progression defects [125]. In turn, this led to decreased cell proliferation through G1 cell cycle arrest (functionally summarized in Table 1).

## 4. Conclusions and Outlook

Long noncoding RNAs are now widely recognized as contributing factors which play diverse and complex roles in cancer. Moreover, they are gaining increasing attention as potential biomarkers and represent a novel class of target molecules. However, we are only beginning to understand the complexity of tumorigenic processes and the role of lncRNAs in melanoma as well as in other cancer types. The clinical integration of lncRNAs as prognostic and predictive biomarkers in conjunction with additional cancer targets, could provide a chance to increase the therapeutic benefit.

Nevertheless, several challenges lie ahead of us. First of all, we have to learn more about the molecular mechanisms and processes employed and controlled by lncRNAs. This task is not trivial and requires multiple techniques as well as the development of novel methods that allow us to observe and capture the lncRNAs and their respective interaction partners and sites in a highly specific manner. For example, in addition to our current understanding that lncRNAs exert their biological effect through interaction with DNA/RNA or proteins, it might be possible that lncRNAs directly interact with other metabolites, such as lipids or sugars [126]. Identifying and studying these lncRNAs will require novel technologies and an open mind.

Secondly, the in vivo function of lncRNAs is difficult to study, due to the low conservation of most lncRNAs [127]. Additionally, multiple strategies have to be considered to generate knockout mouse models for lncRNAs [128]. Once established, these models should be combined with melanoma mouse models to investigate the functional relevance and molecular mechanisms of the lncRNA under investigation.

Finally, these novel animal models could represent valuable tools to develop effective therapeutic reagents against lncRNAs, which are currently considered difficult to target. Reducing the expression or blocking the function of oncogenic lncRNAs with, e.g., small molecules, might pave the way to novel treatment strategies and their clinical application in the future.

## Figures and Tables

**Table 1:** Overview of lncRNA function(s) in melanoma.

LncRNA Name	Function	References
ANRIL	Represses the transcription of *CDKN2A/B* which leads to perturbation in the cell cycle, increased migration and colony formation.	[94,95,96]
BANCR	High levels of BANCR lead to increased migration (by targeting CXCL11) and proliferation. High levels of BANCR directly correlated with tumor stage and indirectly with survival.	[97,98]
CASC15	Promotes melanoma progression and invasiveness. Direct correlation between tumor stage and expression levels.	[99]
GAS5	Indirectly correlates with melanoma migration and invasiveness over reduced levels of MMP2.	[101]
HOTAIR	HOTAIR is up-regulated in metastases compared to the primary tumor, favoring a pro-metastatic role.	[102]
Llme23	Llme23 promotes the expression of the proto-oncogenic RAS-related small GTPase Rab23.	[106]
MALAT1	Possibly involved in cell proliferation and invasion. It does this by targeting miR-22 in cutaneous melanoma and miR-140 in uveal melanoma.	[109,112,113]
PAUPAR	It is a tumor suppressor lncRNA and reduces cell migration and metastases.	[114]
RMEL3	Depletion led to decreased cell survival and proliferation in *BRAF^V600E^* melanoma cell lines.	[116]
SAMMSON	Promotes cell viability and growth irrespective of melanomas mutational status.	[117]
SNGH5	Increased serum levels in patients with melanoma.	[119]
SPRTY4-IT1 (SPRIGHTLY)	Associated with melanoma-genesis; Associated with higher tumor stage and worse prognosis.	[122,123]
UCA1	Promotes invasion and cell proliferation.	[109,125]

## Abbreviations

AMPK	adenosine monophosphate-activated protein kinase
ANRIL	antisense lncRNA in INK4 locus
BANCR	BRAF-activated non-coding RNA
BRAF	B-Raf proto-oncogene, serine/threonine kinase
BRIM-3	BRAF Inhibitor in Melanoma 3
CASC15	cancer susceptibility candidate 15
CDKN2A	cyclin dependent kinase inhibitor 2A
ceRNA	competing endogenous RNA
COOLAIR	cold induced long antisense intragenic RNA
CTLA-4	cytotoxic T-lymphocyte-associated protein 4
DTIC	dacarbazine
ENCODE	ENCyclopedia of DNA Elements
ERK	extracellular signal-regulated kinase
GAS5	growth arrest-specific transcript 5
HES1	hairy and enhancer of split-1
HOTAIR	HOX transcript antisense RNA
HOTTIP	HOXA distal transcript antisense RNA
HOX	homebox
HOXA	homebox A
KIT	KIT proto-oncogene receptor tyrosine kinase
lncRNA	long non-coding RNA
LSD1	lysine-specific histone demethylase 1
mAb	monoclonal antibody
MALAT1	metastasis-associated lung adenocarcinoma transcript 1
MAPK	mitogen-activated protein kinase
MEK	MAPK/ERK Kinase
miRNA	microRNA
MITF	melanogenesis associated transcription factor
MMP	matrix metalloproteinase
NBR2	lncRNA neighbor of BRCA1 gene 2
ncRNA	non-coding RNA
NEAT	nuclear-enriched transcript
NF1	neurofibromin 1
NRAS	neuroblastoma RAS viral (v-ras) oncogene homolog
nt	nucleotides
PAUPAR	PAX6 upstream antisense RNA
PD-1	programmed cell death 1
PD-L1	programmed cell death ligand-1
piRNA	PIWI-interacting RNA
PRC	polycomb repressive complexe
PSF	protein associated splicing factor
RAF	rapidly Accelerated Fibrosarcoma
RAS	rat sarcoma
RTK	receptor tyrosine kinase
SAMMSON	survival associated mitochondrial melanoma-specific oncogenic non-coding RNA
siRNA	small interfering RNA
SNHG5	SnoRNA host gene 5
SPRY4-IT1	SPRY4 intronic transcript 1
TERT	telomerase reverse transcriptase
TIM-3	T-cell immunoglobulin and mucin-domain containing-3
TP53	tumor protein p53
UCA1	urothelial carcinoma-associated 1
UV	ultraviolet
